# RNA Panel Sequencing Is an Effective Tool to Help Classify Splice Variants for Clinical Oncogenetic Diagnosis

**DOI:** 10.1155/2024/4830045

**Published:** 2024-04-02

**Authors:** Maud Privat, Flora Ponelle-Chachuat, Sandrine Viala, Nancy Uhrhammer, Mathis Lepage, Anne Cayre, Yannick Bidet, Yves-Jean Bignon, Mathilde Gay-Bellile, Mathias Cavaillé

**Affiliations:** ^1^Université Clermont Auvergne, INSERM, U1240 Imagerie Moléculaire et Stratégies Théranostiques, Clermont-Ferrand, France; ^2^Département d'Oncogénétique, Centre Jean Perrin, Clermont-Ferrand, France; ^3^Département de Pathologie, Centre Jean Perrin, Clermont-Ferrand, France

## Abstract

Routine gene panel analysis identifies pathogenic variants in clinically relevant genes. However, variants of unknown significance (VUSs) are commonly observed, many of which potentially have an impact on mRNA transcription and splicing. Several software programs attempt to predict the impact of variants on splicing and thus make it possible to select the variants for which it is important to study the effect on the transcripts. Transcript analysis is also necessary to show the tandem character of large duplications, and it can be useful for the search for deep intronic variants that are difficult to identify in a DNA panel. We analyzed 53 variants of unknown significance by targeted sequencing of 48 genes using RNA extracted from patient blood samples. RT-PCR and Sanger sequencing of patient mRNA or minigene monoallelic analysis was also carried out when necessary. For the 53 VUSs, 21 could be classified as likely neutral and 10 as pathogenic or likely pathogenic. Data are comprehensively presented for four variants: *PTEN* c.206+6T>G, *MLH1* c.791-489_791-20del, *BRCA2* c.68-8_68-7delinsAA, and *MSH2* c.(1076+1_1077-1)_(1276+1_1277-1)dup. These four examples illustrate the usefulness of blood RNA panel sequencing in clinical oncogenetics to help classify VUSs with predicted splice effects. It could also be useful for characterizing large duplications and for detecting deep intronic variants with an impact on expressed transcripts.

## 1. Introduction

Oncogenetics is aimed at stratifying the risk of cancer in the population, in order to offer appropriate monitoring. For this, pathogenic variants in cancer predisposition genes are sought by sequencing and comparison to the reference sequence of these genes. With the deployment of NGS methods, the number of genes analyzed for each patient has increased considerably in clinical routine. Thus, very many variants in these genes are identified, for which it is essential to determine whether they are pathogenic or neutral for the function of the protein. A classification system has been proposed by Plon et al. [1]. The American College of Medical Genetics and Genomics then published recommendations for variant classification that are widely used in oncogenetic laboratories [2]. This makes it possible to effectively classify all variants identified by NGS analysis, according to the current knowledge, but the majority unfortunately remain variants of unknown significance (VUSs). Indeed, 15-25% of patients who underwent cancer multigene panel testing are found to carry at least one VUS, depending on the genes tested [3, 4].

Among these VUSs, an important proportion are predicted to have an impact on splice mechanisms by various splice prediction software programs [5, 6]. For example, Karam et al. studied 307,812 patients that underwent multigene cancer panel testing [4]. They found 52,831 patients (17%) with 15,859 unique VUSs, including 1,672 variants with predicted splicing impact (10.5%). Moreover, some hereditary cancer genes may be enriched in splicing mutations [7]. Prediction of splicing impact on every variant is thus crucial when analyzing diagnostic DNA panel sequencing. These algorithms make it possible, when the prediction is negative, to exclude an impact on the splicing of variants of unknown significance. False negatives are relatively rare (negative predictive value > 95% for SpliceAI, for example, [8]), which often makes it possible to classify these variants as likely benign. However, a relatively high rate of false positives is reported: for example, *in silico* models yielded a 25% false-positive rate in Karam et al.'s study on 64 variants [4]. Wai et al. also reported positive predictive values between 46 and 83% depending on the software used, in a study of 257 experimentally validated variants [8]. A positive prediction therefore does not make it possible to classify VUSs, but it encourages further investigations on RNA. Thus, different methods for studying RNA have been reported, but they all can be difficult to apply in routine diagnostics [9]. Here, we present a multigene capture approach to study transcripts of targeted genes. This technique is very close to the multigene panel techniques traditionally performed in constitutional genetics laboratories. It is therefore easily applicable in diagnostic laboratories, and it can still be supplemented by other techniques if necessary, such as RT-PCR or minigenes.

Since 2016, 5,113 patients with hereditary predisposition to cancer were analyzed by multigene panel sequencing in the oncogenetics department of Centre Jean Perrin. 3,766 variants of unknown significance were identified, including 450 VUSs (12%) with positive splice prediction. This study presents our RNA panel analyses on 53 different VUSs in order to evaluate the efficiency of the method to classify splice variants.

## 2. Material and Methods

### 2.1. Ethical Approval

All patients signed an informed consent for the use of their samples for research purposes. The study was approved by an ethics committee (CPP Sud-Est VI: 2023/CE18).

### 2.2. Selection of Splice Variants

Between 2016 and 2022, 5,113 patients consulting the oncogenetics department of the Centre Jean Perrin underwent hereditary cancer predisposition panel analysis, according to national or international recommendations where available, or according to data from literature (Table 1). All variants identified on this DNA panel were subjected to the SpIP and SpliceAI prediction algorithms. Fifty-three VUSs were selected for an RNA panel, during multidisciplinary meetings based on the clinical presentation of the families and the role of the gene:

- 20 variants with high splicing predictions (>50%) according to SpIP and/or SpliceAI
- 18 variants with moderate splicing predictions (20 to 50%) according to SpIP and/or SpliceAI
- 2 suspicious variants without available SpIP and/or SpliceAI predictions
- 13 variants without splice predictions (<20%) according to SpIP and/or SpliceAI

In addition, four samples were included to search for deep intronic variants (patients presenting a severe clinical phenotype (Lynch syndrome, for example) but no constitutional pathogenic variant identified by the DNA panel). Finally, four other samples with exon duplication identified by the DNA panel were also included for duplication characterization.

### 2.3. Targeted Panel on Peripheral Blood RNA

Peripheral blood was collected in PAXgene blood RNA tubes, after informed consent was obtained from each patient. Total RNA was isolated using the PAXgene Blood RNA kit (Qiagen, Courtaboeuf, France). Screening for transcript abnormalities was performed by sequencing a panel of 48 genes associated with hereditary cancer syndromes (Table 1). Libraries were prepared using the KAPA RNA Hyper Prep kit (Roche, Mannheim, Germany). Sequences of interest were then captured with a custom design of Nimblegen SeqCap EZ Choice or Hypercap (Roche, Mannheim, Germany) and sequenced on a MiSeq or Nextseq 550 instrument (Illumina, San Diego, USA).

Reads were aligned to the human reference genome (genome assembly GRCh37) using STAR aligner v2.7.10a (Spliced Transcript Alignment to a Reference) [10]. SpliceLauncher was used to compute a junction read count matrix. A list of transcripts to use as reference is given to SpliceLauncher to compute the relative expression over natural junctions and detect abnormally expressed junctions [11].

### 2.4. RT-PCR Analysis of Peripheral Blood RNA

RNA was reverse-transcribed using oligo(dT) primers with the Superscript III First-Strand Synthesis System for RT-PCR (Life Technologies, Saint Aubin, France), and cDNA was amplified using two different pairs of primers located around the predicted splice effect. RT-PCR products were separated by electrophoresis both on an Agilent Bioanalyzer DNA1000 chip (Agilent, Les Ulis, France) and on an agarose gel. After purification using Agencourt Ampure XP (Beckman Coulter, Villepinte, France) or the MinElute PCR Purification kit (Qiagen, Courtaboeuf, France), RT-PCR products were sequenced by using the BigDye Terminator kit (Fisher Scientific, Illkirch, France).

### 2.5. Minigene Splicing Assay

A splicing reporter minigene assay of some variants was performed using the pCAS2 vector, as described [12]. Exons or introns in which the variants are located were PCR amplified from patients' genomic DNA using the FastStart High Fidelity PCR System dNTP Pack v7 (Roche, Mannheim, Germany) and forward and reverse primers carrying restriction sites for BamH1 and MluI, respectively. PCR products were cloned into the pCAS2 vector. All constructs were verified by Sanger sequencing using the BigDye Terminator kit (Fisher Scientific, Illkirch, France). Wild-type and mutant constructs were transfected into HeLa cells. Cells were harvested after 24 h, and total RNA was extracted using the RNeasy mini kit (Qiagen, Courtaboeuf, France). Reverse transcription was performed using the Superscript III First-Strand Synthesis System for RT-PCR (Life Technologies, Saint Aubin, France) following the manufacturer's instructions. cDNA was amplified with AmpliTaq DNA polymerase (Fisher Scientific, Illkirch, France) using pCAS-KO1-F (5′-TGACGTCGCCGCCCATCAC-3′) and pCAS-2R (5′-ATT GGTTGTTGAGTTGGTTGTC-3′) as forward and reverse primers, respectively. PCR products were separated on an Agilent Bioanalyzer DNA1000 chip (Agilent, Les Ulis, France). Each PCR product was purified using Agencourt Ampure XP (Beckman Coulter, Villepinte, France) and sequenced using the BigDye Terminator kit (Fisher Scientific, Illkirch, France).

### 2.6. Immunohistochemistry

PTEN expression was determined by IHC on 3 *μ*m paraffin sections with the PTEN (D4.3) XP rabbit monoclonal antibody (Cell Signaling Technology, Beverly, MA, United States). Antigen retrieval was carried out for 90 min in CC1 buffer on a Benchmark-ULTRA immunostainer (Roche, Mannheim, Germany). The antibody was incubated for 1 hour at 1/125 dilution at room temperature, and the revelation was done with the Ultraview DAB kit (Roche, Mannheim, Germany). The signalSlide PTEN IHC control slide is used to validate the technique.

## 3. Results

We performed targeted blood RNA sequencing to help classify 53 different VUSs with potential splicing impact (Table 2). Several biological or technical replicates were carried out to test the intra- and intersample reproducibility of the technique. Two variants were analyzed for several patients: *BRCA2* c.6842-8_6842-7del was analyzed for three different patients and *NF2* c.1122+6T>C for two patients. The results observed were very similar regardless of the patient. For eight other variants, technical replicates of RNA libraries and targeted sequencing were performed, showing good reproducibility of the method (data not shown). For all variants for which an effect on splicing was demonstrated by the RNA panel, this effect was verified by RT-PCR and Sanger sequencing, except for *CDH1* c.1901C>T because the splice effect observed was already published [13]. For eight variants without splice effect on the RNA panel, we also carried out RT-PCR and Sanger sequencing to confirm the absence of any impact on splicing. Finally, for three variants of particular clinical importance, monoallelic minigene analysis was also performed in order to check the partial effect or NMD implication.

**Figure 1:**
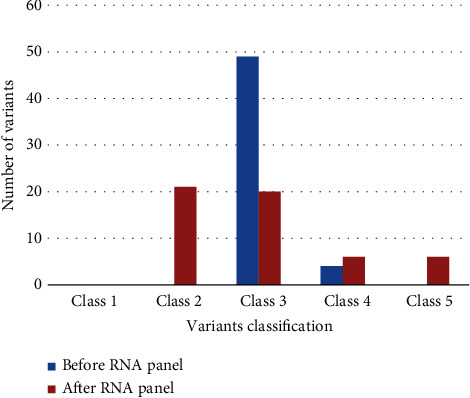
Variant classification before and after RNA panel sequencing.

For the 53 VUSs analyzed, 20 (37.7%) induced partial or total modification of the transcript and 10 variants could be classified as pathogenic or likely pathogenic (Figure 1). For the other 10 variants, either a partial effect on splicing or in-frame exon skipping was observed, which did not make it possible to conclude on pathogenicity. Among the 33 VUSs that did not show an impact on splicing, 21 could be classified as likely neutral. For six variants, no abnormal transcripts were observed, but in the absence of any heterozygous exonic variant to verify the presence of the two alleles and exclude allele dropout by nonsense-mediated decay, these remained VUSs. For the last six variants, we were able to conclude that there was no effect on splicing, but these were missense variants for which the functional impact of the amino acid modification was not known.

We compared the performance of two popular splicing prediction software programs: SpliceAI [14] and SpIP [6] (Table 3). SpIP is a random forest model running a cascade of bioinformatics tools. Briefly, SPiP uses a SPiCE tool for the consensus splice sites (donor and acceptor sites), MES for the polypyrimidine tract between -13 and -20, BPP for the branch point area between -18 and -44, a homemade score to reveal cryptic/de novo activation, and *Δ*tESRseq for exonic splicing regulatory elements up to 120 nt from the exon boundaries. SpliceAI is a deep neural network that accurately predicts splice junctions from an arbitrary pre-mRNA transcript sequence. Considering all positive predictions regardless of score, we found better sensitivity for SpliceAI than SpIP (81% vs. 47%) but a slightly lower specificity (94% vs. 100%) (Table 3). Focusing on variants with splicing altering predictions greater than 50%, the sensitivity rises to 79% for SpIP and 92% for SpliceAI. Most of the positive predictions with SpIP but negative with SpliceAI were for predictions below 50%, for which no impact on splicing was demonstrated by the RNA panel. Only two variants (*NF2* c.1122+6T>C and *MLH1* c.882C>G) were negative with SpliceAI and highly positive (>90%) with SpIP. Our RNA panel showed no splice impact for the *NF2* variant but a partial exon 10 skipping of *MLH1* for c.882C>G. Finally, one variant (*PALB2* c.2379C>T) was negative for SpIP but highly positive for SpliceAI (66%) but gave no abnormal transcripts in our RNA panel. The 13 variants with negative predictions for both algorithms showed no impact on transcripts in our RNA panel.

In addition to the 53 VUSs studied for their impact on splicing, we analyzed the RNA panel in two other situations: the search for deep intronic variants and the characterization of large tandem duplications. For samples with a severe clinical phenotype (Lynch-like syndrome, for example) but no constitutional pathogenic variant identified using DNA, we tested if RNA could show abnormal transcripts, suggesting a pathogenic deep intronic variant. Four patients with a suggestive clinical phenotype but without mutations found on the DNA panel were tested: one patient showed a severe breast cancer family and three patients developed Lynch syndrome spectrum tumors with protein expression profiles suggestive of a mutation in an MMR gene. For one of them, a deep intronic variant could be demonstrated (*MLH1* c.791-489_791-20del, see the specific paragraph on this variant). Finally, we used targeted RNA sequencing to characterize large duplications. We tested four samples with duplications of at least one exon to assess whether the duplication was in tandem (Table 4). For three cases, chimeric reads proved the duplication in tandem, allowing reclassification of these duplications as pathogenic.

Four examples are presented to illustrate the utility of this approach: two analyses (*PTEN* c.206+6T>G and *BRCA2* c.68-8_68-7delinsAA) illustrate complex or partial splicing effects that required complementary studies, one example of successful deep intronic variant search (*MLH1* c.791-489_791-20del) and one example of duplication characterization (*MSH2* c.(1076+1_1077-1)_(1276+1_1277-1)dup).

### 3.1. *PTEN* c.206+6T>G

**Figure 2:**
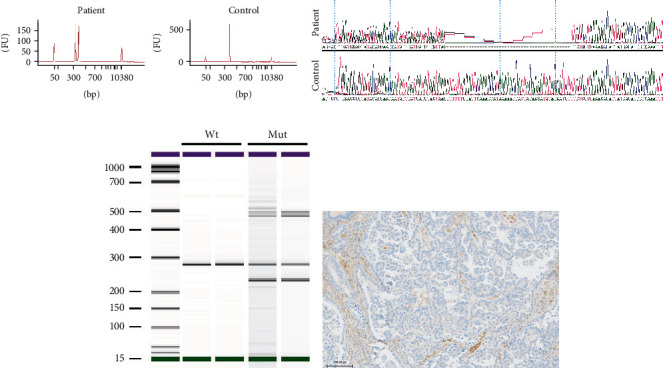
Study of the *PTEN* c.206+6T>G variant. (a) RT-PCR analysis on blood sample RNA: peripheral blood of the patient with the *PTEN* c.206+6T>G variant was collected in PAXgene blood RNA tubes. RT-PCR analysis was performed with primers mapping to exons 2 and 5, and PCR products were separated by bioanalyzer electrophoresis. The 370 bp peak corresponds to the reference *PTEN* transcript, and the 325 bp peak corresponds to a *PTEN* transcript lacking exon 3. RT-PCR products were then analyzed by Sanger sequencing. (b) Minigene analysis: HeLa cells were transfected with pCAS2 vectors including wild-type or mutant *PTEN* sequences. Total RNA was isolated, RT-PCR analysis was performed using pCAS primers, and PCR products were separated by bioanalyzer electrophoresis. The 280 bp band corresponds to the reference *PTEN* transcript, and the 235 bp band corresponds to a *PTEN* transcript lacking exon 3. (c) PTEN immunohistochemistry: PTEN expression of the endometrium tumor was determined by immunohistochemistry on 3 *μ*m paraffin sections with a PTEN rabbit antibody (Cell Signaling Technology). The PTEN IHC control slide was used to validate the technique.

A 39-year-old woman presenting clear cell papillary adenocarcinoma of the endometrium was seen in an oncogenetics consultation at the Jean Perrin Center. She was thyroidectomized at the age of 26 for a multihetero nodular thyroid with elevated calcitonin; examination of this thyroidectomy did not find C-cell hyperplasia or medullary carcinoma, but several adenomas were found on both lobes, and there was a small oncocytic adenoma in the left lobe. Panel sequencing of blood DNA revealed the intronic variant c.209+6T>G in the *PTEN* gene. This variant is predicted by both SpIP and SpliceAI to impact the consensus splice site of exon 3 (Table 2). The skipping of exon 3 of the *PTEN* gene results in the loss of 16 amino acids within the phosphatase domain and is recognized as pathogenic [15]. Analysis of RNA extracted from peripheral blood by panel sequencing showed equal depths of full-length and exon 3-omitted transcripts, revealing that the skipping of exon 3 is total in the altered allele. The same result was observed by RT-PCR of the same RNA sample (Figure 2(a)), using primers specifically amplifying the *PTEN* cDNA and not its pseudogene. There was no exonic variant present to verify the absence of a normally spliced product for the variant allele. A monoallelic splicing test by minigene resulted in major but partial exon 3 skipping (Figure 2(b)). These contradictory results do not allow us to conclude on a complete or partial effect. This may be due to a differential impact of the variant on splicing depending on the tissue. Nevertheless, the mother of this patient, who carries the *PTEN* variant, developed breast cancer at 65 years old and underwent partial thyroid surgery at ages 27 and 39 for multiheteronodular goiter. Moreover, immunohistochemistry on the endometrial tumor of this patient showed a complete loss of PTEN protein expression (Figure 2(c)). Overall, we classified this variant as likely pathogenic.

### 3.2. *MLH1* c.791-489_791-20del

**Figure 3:**
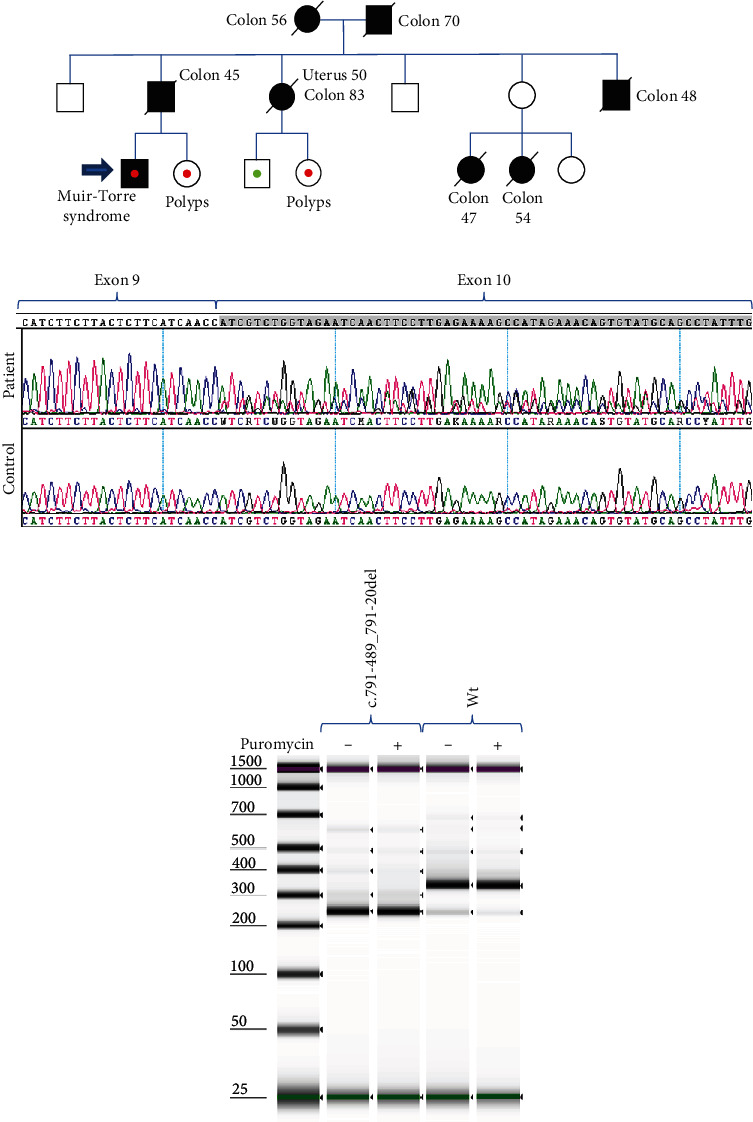
Study of the *MLH1* c.791-489_791-20del variant. (a) Pedigree: fill symbols indicate patients affected with cancer. Open symbols indicated relatives unaffected with cancer. The type of cancer and age at presentation are given under the symbol. Red dots indicate carriers of the *MLH1* variant; green dots indicate people who do not carry the *MLH1* variant. (b) RT-PCR analysis on blood RNA: peripheral blood of the patient with the *MLH1* c.791-489_791-20del variant was collected in PAXgene blood RNA tubes. RT-PCR analysis was performed with primers forward and reverse mapping to exons 8 and 11, respectively, and PCR products were separated by bioanalyzer electrophoresis. RT-PCR products were then analyzed by Sanger sequencing. (c) Minigene analysis: HeLa cells were transfected with wild-type or mutant plasmids. Total RNA was isolated, RT-PCR analysis was performed, and PCR products were separated by bioanalyzer electrophoresis. The 338 bp band corresponds to the reference *MLH1* transcript, and the 244-bp band corresponds to a *MLH1* transcript lacking exon 10.

A 55-year-old man diagnosed with Muir-Torre syndrome consulted our oncogenetics department. Loss of nuclear labeling for the MLH1 protein was observed in a sebaceous adenoma. Several cases of colon cancer have been identified in the family (Figure 3(a)). Panel sequencing of blood DNA did not identify any variant in the MMR genes. RNA panel analysis looking for deep intronic variants revealed partial skipping of *MLH1* exon 10. Major but not total exon 10 skipping was confirmed by RT-PCR and Sanger sequencing of exons 9-11 of MLH1 (Figure 3(b)). New analysis of our DNA panel with the DELLY tool [16] identified a large deletion in intron 10 of *MLH1* c.791-489_791-20del. This deletion is predicted by SpIP to impact splice mechanisms (alter BP + alter by creating cryptic 36.17% (26.46%-45.88%)). No heterozygous exonic variant was present in *MLH1* in the constitutional DNA to confirm transcription of the 2 alleles. Thus, it is possible that a part of the aberrant transcript was degraded by NMD, explaining the partial exon skipping observed. To check this hypothesis, we performed a monoallelic test by minigene treated or not with puromycin, an NMD inhibitor. The results show total exon 10 skipping with the *MLH1* c.791-489_791-20del plasmid and very partial exon 10 skipping with the wild-type *MLH1*, regardless of puromycin treatment (Figure 3(c)). In addition, a cosegregation study showed that two carriers of the variant have developed colorectal polyps and two obligate carriers developed colon and/or uterine cancer (Figure 3(a)). Altogether, we consider this variant to be likely pathogenic.

### 3.3. *BRCA2* c.68-8_68-7delinsAA

A woman with breast cancer at age 49 and pancreatic cancer at age 71 was seen in our oncogenetics consultation. Her brother had prostate cancer at age 76. DNA panel sequencing identified the *BRCA2* c.68-8_68-7delinsAA variant, which weakens the acceptor splice site according to SpIP prediction algorithms (alter by Spice 69.57% (61.89%-77.25%)). Analysis of the patient's blood RNA by panel sequencing showed partial skipping of *BRCA2* exon 3 (r.68_316del, 42% of the variant-carrying allele). Because of the partial effect observed and the low reading depth of *BRCA2* (due to the low expression of this gene in lymphocytes), we analyzed the RNA panel for this sample in triplicate (different libraries and different sequencing runs). We confirmed exon 3 skipping for 42 to 82% of the variant-carrying allele. Exon 3 is in-frame, but complete exon 3 skipping has been proven to be pathogenic [17]. The partial effect of c.68-8_68-7delinsAA has been described by other techniques (fragment analysis and competitive Q-PCR) [18, 19]. In our RNA panel, we also observed another minor transcript with skipping of exon 3 + 4 bases of exon 4 (r.68_320del, between 0 and 25% of the variant-carrying allele, depending on the replicate). This transcript is not predicted by the algorithms and has therefore not been studied by published targeted methods. RT-PCR and Sanger sequencing with primers in exons 2 and 6 confirmed the major exon 3 skipping, but we could not detect the minor r.68_320del transcript (if present), potentially due to insufficient sensitivity of this technique. These data are not sufficient to conclude on the pathogenicity of the *BRCA2* c.68-8_68-7delinsAA variant and will have to be supplemented by minigene analysis including exons 3 and 4 of *BRCA2*. Long-read sequencing could also help to understand the impact on several exons. Finally, this variant is included in the French cosegregation study COVAR [20], in order to progress on its clinical significance.

### 3.4. *MSH2* c.(1076+1_1077-1)_(1276+1_1277-1)dup

**Figure 4:**
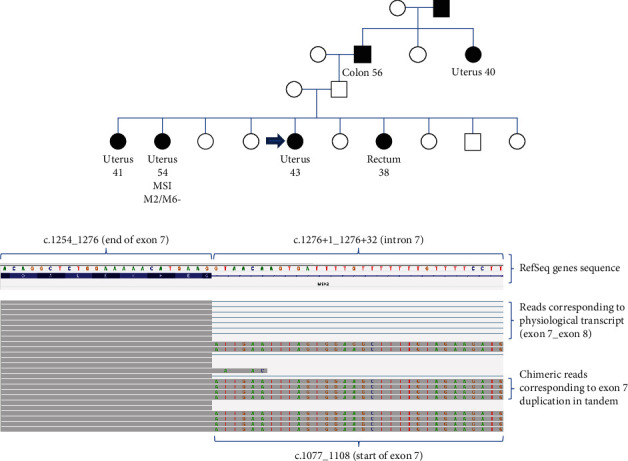
Study of the *MSH2* c.(1076+1_1077-1)_(1276+1_1277-1)dup variant. (a) Pedigree: filled symbols indicate patients affected with cancer. Open symbols indicated relatives unaffected with cancer. The type of cancer and age at presentation are given under the symbol. MSI: tumoral microsatellite instability; M2/M6-: tumoral extinction of MSH2 and MSH6 protein expression (seen by immunohistochemistry). (b) Panel sequencing on blood sample RNA: peripheral blood of the patient with the *MSH2* exon 7 duplication was collected in PAXgene blood RNA tubes. Targeted panel sequencing was performed using KAPA kits and probes on the Illumina device. Sequences were visualized and manually analyzed using Integrated Genomics Viewer (Broad Institute) software.

A 43-year-old woman presented with endometrioid adenocarcinoma. Her siblings were affected with cancer of the uterus (at 41 and 54 years old) and rectal cancer at 38 years old. Her grandfather had colon cancer at age 56, and a paternal great-aunt presented with cancer of the uterus at age 40 (Figure 4(a)). One of the uterine cancers presented microsatellite instability and loss of MSH2 and MSH6 protein expression. The analysis of MMR genes by Sanger sequencing did not reveal any pathogenic variant, but this family is still suspected of Lynch syndrome. Thus, a constitutional mutation in hereditary colon cancer genes was investigated by DNA panel sequencing. Duplication of *MSH2* exon 7, c.(1076+1_1077-1)_(1276+1_1277-1)dup, was identified. Short-read DNA sequencing cannot distinguish whether this duplication is in tandem (and is therefore pathogenic because it alters the reading frame) or whether the extra copy of exon 7 is inserted elsewhere in the genome (and therefore does not alter the transcription of the *MSH2* gene). Our panel of blood-extracted RNA offered a quick and easy response to this question as we could directly observe the *MSH2* transcripts that contained the exon 7 repeat (Figure 4(b)). We concluded that the duplication of *MSH2* exon 7 is pathogenic in this family.

## 4. Discussion

Multigene panel sequencing of total RNA extracted from peripheral blood was performed to study the splice impact of variants on transcripts. This technique is easy to implement in a routine oncogenetics laboratory and allows direct observation of aberrant transcripts. Unlike RT-PCR, it is a technique without a priori, so there is no need to start with a fixed hypothesis about how the modified transcript is structured. Of the 53 VUSs studied, 10 could be classified as pathogenic or likely pathogenic, due to their impact on splicing as highlighted by the RNA panel. Twenty-one intronic or synonymous variants could be classified as probably neutral, as the RNA panel showed no impact on splicing, and it is therefore very unlikely that these silent variants modify protein function. For six missense variants, an effect on splicing could be excluded, although this did not change their class, since an impact of the amino acid modification could not be excluded. Our RNA panel enabled us to modify the classification of 58% of the variants studied (31/53). Karam et al. reported an 86% rate of variants classified by RNA genetic testing (55 of 64 variants) [4]. Wai et al. studied 257 variants by RT-PCR analysis and found 85 variants (33%) associated with abnormal sequencing [8]. These results on the reclassifying rate facilitated by RNA analysis vary greatly depending on the choice of variants tested. Although SpliceAI showed a better sensitivity than SpIP in our results, one variant (*MLH1* c.882C>G) caused a partial exon 10 skipping that was predicted by SpIP but not by SpliceAI. Moreover, SpIP was already shown to have better performance than SpliceAI in the branch point area and in exonic regions [6]. All variants with negative predictions with both algorithms were confirmed to have no impact on the transcripts. In the future, we have therefore chosen to classify synonymous or intronic variants with no prediction of splicing as probably neutral, without RNA studies. Variants with moderate predictions (between 20 and 50% with SpIP and/or SpliceAI) can be studied by RNA panels with a good chance of classifying when a heterozygous exonic variant is present to allow observation of both alleles. However, few of these variants showed an impact on splicing (2/17, 11%). For our subsequent RNA panel analyses, we therefore decided to systematically study only variants with strong splicing predictions (SpIP and/or SpliceAI).

The variants that remained VUSs illustrate the different limits of the technique. The first limit is the difficulty evaluating nonsense-mediated mRNA decay (NMD) if no heterozygous exonic variant is present. This problem could be solved by working with lymphoblastoid lines, which can be treated with puromycin to inhibit NMD. Otherwise, monoallelic tests with a minigene system can be used with puromycin, but these techniques require cell culture equipment. RNA panel and minigene analyses are not mutually exclusive and could therefore be used successively: an RNA panel could be performed as part of routine diagnostics, while minigene analyses could be performed only when the RNA panel is unable to determine the pathogenicity of certain clinically important variants.

Concluding on the pathogenicity of a variant may also be complicated by the observation of partial effects on splicing and aberrant splicing that preserves the reading frame. Recent recommendations have been published by the ClinGen Splicing Subgroup to help classify splice variants according to the ACMG framework [21]. Physiological alternative splicing events have been described for many predisposition genes, and they can help the interpretation of VUS splicing impact [22–27]. Nevertheless, functional studies remain necessary to advance on the classification of variants with partial or in-frame splicing effects.

Another limitation of this blood RNA panel is its dependence on the expression of genes of interest in lymphocytes. *BRCA2*, for example, is poorly expressed in blood, although we did obtain sufficient depth of coverage. Moreover, variant classification based on blood RNA panel results should be performed with caution, especially if the observed splicing is normal. Indeed, the effects on splicing could be different on the target tissues [28]. To our knowledge, alternative splicing of breast predisposition genes seems to be similar in blood and breast tissues [26, 27], suggesting that the observed results are pertinent for evaluating the associated risks.

For most variants, the RNA panel alone made it possible to answer the question of the effect on splicing. RT-PCR can be used to confirm the quantification of the different transcripts by another technique (in the event of a partial effect, for example). It can also be useful for low-expressed genes. Minigene is a monoallelic test with the possibility of treatment with puromycin. It can be used when degradation by NMD cannot be excluded in the RNA panel. Both techniques are therefore still necessary for the partial or complex splicing effects observed in the RNA panel.

This RNA sequencing panel may also be of interest for characterizing large duplications. Four RNA analyses were performed for patients with one or two exon duplications detected on gDNA panel analyses. For two of them, it was possible to observe a tandem duplication of the exons and to conclude that the variants were pathogenic. For the duplication of exons 11 and 12 of *PMS2*, we could not conclude because of the very homologous pseudogene in this region. For the last patient with exon duplication, we did not find any reads showing tandem duplication, probably because the exon duplication was elsewhere in the genome.

Finally, RNA panel sequencing could likely be used to identify the production of aberrant transcripts due to deep intronic variants not detected by classical DNA panel analyses. Only four analyses of this type were carried out in our study, one of which identified the *MLH1* c.791-489_791-20del variant in a typical Lynch syndrome family. We believe that these RNA analyses could be offered for patients with a strong family history suggesting a genetic predisposition to cancer but without a pathogenic variant found on the DNA panel.

As a conclusion, blood RNA panel sequencing is an easy technique to implement in an oncogenetics laboratory, and it was revealed to be an efficient tool to help classify VUSs with predicted splice effect. It could also be useful for characterizing large duplications and for researching deep intronic variants' impact on expressed transcripts. Nevertheless, it provides only an argument in favor or not of the pathogenicity of the variants, which must be interpreted with caution, especially for partial effects or for low-expressed genes.

## Figures and Tables

**Table 1:** List of the 48 genes sequenced in the CJP familial cancer panel.

Gene	Reference
*AIP*	NM_003977.4
*APC*	NM_000038.6
*ATM*	NM_000051.4
*BAP1*	NM_004656.4
*BMPR1A*	NM_004329.3
*BRCA1*	NM_007294.4
*BRCA2*	NM_000059.4
*BRIP1*	NM_032043.3
*CASR*	NM_000388.4
*CDC73*	NM_024529.5
*CDH1*	NM_004360.5
*CDK4*	NM_000075.4
*CDKN2A*	NM_000077.5
*CHEK2*	NM_007194.4
*EPCAM*	NM_002354.3
*FH*	NM_000143.4
*FLCN*	NM_144997.7
*MAX*	NM_002382.5
*MC1R*	NM_002386.4
*MEN1*	NM_130799.2
*MET*	NM_001127500.3
*MITF*	NM_000248.4
*MLH1*	NM_000249.4
*MSH2*	NM_000251.3
*MSH6*	NM_000179.3
*MUTYH*	NM_001048174.2
*NBN*	NM_002485.5
*NF1*	NM_000267.3
*NF2*	NM_000268.4
*PALB2*	NM_024675.4
*PMS2*	NM_000535.7
*POLD1*	NM_001256849.1
*POLE*	NM_006231.4
*PTEN*	NM_000314.8
*RAD51C*	NM_058216.3
*RAD51D*	NM_002878.4
*RET*	NM_020975.6
*SDHA*	NM_004168.4
*SDHAF2*	NM_017841.4
*SDHB*	NM_003000.3
*SDHC*	NM_003001.5
*SDHD*	NM_003002.4
*SMAD4*	NM_005359.6
*STK11*	NM_000455.5
*TMEM127*	NM_017849.4
*TP53*	NM_000546.6
*VHL*	NM_000551.4
*WRN*	NM_000553.6

**Table 2:** Summary of the 53 variants studied by our targeted RNA panel.

Gene	Variant	p.	Splice prediction SpIP v2.1	Splice prediction SpliceAI v1.3	Splice effect RNA panel	Splice effect Other criteria	r.	NMD exclusion	ACMG criteria	Variant classification
APC	c.7500G>A	p.(Gln2500=)	Alter by creating de novo splice site 05.56% [01.91%-15.11%]	AG: 0.00; AL: 0.01; DG: 0.00; DL: 0.00	Normal	/	r.7500G>A; p.(Gln2500=)	Heterozygous variant in RNA	PM2, BP7_S	2
ATM	c.2922-2A>G	p.?	Alteration of the consensus splice site 98.41% [91.47%-99.96%]	AG: 0.50; AL: 0.98; DG: 0.00; DL: 0.01	Acceptor site modification	Confirmed by RT-PCR	r.2922_2953del; p.(Asn975Cysfs∗3)	/	PM2, PVS1	4
ATM	c.7896C>T	p.(Asn2632=)	NTR 07.62% [04.42%-12.08%]	AG: 0.00; AL: 0.00; DG: 0.00; DL: 0.01	Normal	Confirmed by RT-PCR	r.7896C>T; p.(Asn2632=)	Heterozygous variant in RNA	PM2, BP4, BP7_S	2
ATM	c.4909+3G>A	p.?	Alteration of the consensus splice site 85.91% [79.27%-91.06%]	AG: 0.00; AL: 0.00; DG: 0.35; DL: 0.14	Normal	Confirmed by RT-PCR	r.=	Heterozygous variant in RNA	PM2, PP3, BP7_S	2
ATM	c.2377-6T>A	p.?	Alteration of the consensus splice site 30.67% [23.41%-38.71%]	AG: 0.04; AL: 0.03; DG: 0.00; DL: 0.00	Normal	/	r.=	/	PM2, BP7_S	2
ATM	c.8671+2_8671+3insTA	p.?	NA	NA	Exon 59 skipping	Confirmed by RT-PCR Co-segregation with breast cancer (29)	r.8585_8671del; p.(Val2862_Leu2890del)	/	PM2, PP1, PVS1	5
ATM	c.7785T>C	p.(Asp2595=)	NTR 05.05% [02.45%-09.09%]	AG: 0.00; AL: 0.00; DG: 0.01; DL: 0.00	Normal	/	r.7785T>C; p.(Asp2595=)	/	PM2, BP7_S	2
ATM	c.2679A>G	p.(Gln893=)	Alteration of an exonic splicing regulatory element 35.81% [28.11%-44.1%]	AG: 0.00; AL: 0.01; DG: 0.00; DL: 0.00	Normal	/	r.2679A>G; p.(Gln893=)	Heterozygous variant in RNA	PM2, BP7_S	2
ATM	c.2124+1G>T	p.?	Alteration of the consensus splice site 98.41% [91.47%-99.96%]	AG: 0.00; AL: 0.00; DG: 0.02; DL: 1.00	Exon 13 skipping	Confirmed by RT-PCR	r.1899_2124del; p.(Cys633∗)	/	PM2, PVS1	4
ATM	c.2839-1G>T	p.?	Alteration of the consensus splice site 98.41% [91.47%-99.96%]	AG: 0.29; AL: 0.89; DG: 0.00; DL: 0.00	Cryptic acceptor site creation	Confirmed by RT-PCR Identified in AT patient	r.2839_2856del; p.(Tyr947_Lys952del)	/	PM2, PVS1_M, PM3	4
ATM	c.8010+30insN[?]	p.?	NA	NA	Partial exon 54 skipping	Confirmed by RT-PCR	p.?	/	PM2, PVS1_NA	3
ATM	c.3078-30_3078-27del	p.?	Alter BP 42.50% [32.25%-53.43%]	AG: 0.03; AL: 0.00; DG: 0.00; DL: 0.00	Normal	/	r.=	No heterozygous variant	PM2, BP7_NA	3
ATM	c.7375C>G	p.(Arg2459Gly)	Alter ESR 35.81% [28.11%-44.1%]	AG: 0.00; AL: 0.00; DG: 0.14; DL: 0.01	Normal	/	r.7375C>G; p.(Arg2459Gly)	Heterozygous variant in RNA	PM2, BP7_NA	3
BAP1	c.720G>A	p.(Lys240=)	NTR 05.46% [04.02%-07.38%]	AG: 0.00; AL: 0.11; DG: 0.00; DL: 0.01	Normal	/	r.720G>A; p.(Lys240=)	Heterozygous variant in RNA	PM2, BP7_S	2
BAP1	c.-9C>A	p.?	Alter ESR 30.18% [25.47%-35.35%]	AG: 0.00; AL: 0.01; DG: 0.00; DL: 0.00	Normal	/	r.=	Heterozygous variant in RNA	PM2, BP7_S	2
BRCA1	c.2518A>T	p.(Ser840Cys)	NTR 01.24% [00.71%-02.15%]	AG: 0.00; AL: 0.00; DG: 0.00; DL: 0.00	Normal	/	r.2518A>T; p.(Ser840Cys)	Heterozygous variant in RNA	PM2, BP7_NA	3
BRCA2	c.9648+1G>A	p.?	Alteration of the consensus splice site 98.41% [91.47%-99.96%]	AG: 0.00; AL: 0.00; DG: 0.01; DL: 1.00	Exon 26 skipping	Confirmed by RT-PCR and mini-gene	r.9502_9648del; p.(Asn3168_Leu3216del)	/	PM2, PVS1_M	3
BRCA2	c.8332-28A>G	p.?	Alteration of the branch point 23.61% [16.94%-31.4%]	AG: 0.00; AL: 0.00; DG: 0.00; DL: 0.00	Normal	Confirmed by RT-PCR	r.=	Heterozygous variant in RNA	PM2, BP7_S	2
BRCA2	c.7670C>T	p.(Ala2557Val)	NTR 07.81 % [04.44%-12.56%] (SpIP v1: alter ESR 28.87% [24.29%-33.93%])	AG: 0.04; AL: 0.02; DG: 0.00; DL: 0.18	Normal	/	r.7670C>T; p.(Ala2557Val)	/	PM2, BP7_NA	3
BRCA2	c.7524C>T	p.(Gly2508=)	NTR 07.8% [04.53%-12.37%] (SpIP v1: alter ESR + alter by creating de novo splice site 28.87% [24.29%-33.93%])	AG: 0.06; AL: 0.00; DG: 0.06; DL: 0.00	Normal	/	r.7524C>T; p.(Gly2508=)	Heterozygous variant in RNA	PM2, BP7_S	2
BRCA2	c.68-8_68-7delinsAA	p.?	NA	NA	Partial exon 3 skipping	Confirmed by RT-PCR	p.?	/	PM2, PVS1_NA	3
BRCA2	c.6842-8_6842-7del	p.?	Alteration of the consensus splice site 98.41% [91.47%-99.96%]	AG: 0.00; AL: 0.43; DG: 0.00; DL: 0.00	Total exon 12 skipping	Confirmed by RT-PCR	r.6842_6937del; p.(Glu2282_Gly2313del)	/	PM2, PVS1_M	3
CDH1	c.1901C>T	p.(Ala634Val)	+ Creation of a new splice site + alteration of an exonic splicing regulatory element 98.41% [91.47%-99.96%]	AG: 0.00; AL: 0.00; DG: 0.91; DL: 0.53	Cryptic donor site creation	Confirmed in bibliography (13)	r.1900_1936del; p.(Ala634Profs)	/	PM2, PVS1, PP5	5
CDH1	c.906C>T	p.(Tyr302=)	NTR 07.8% [04.53%-12.37%]	AG: 0.00; AL: 0.00; DG: 0.01; DL: 0.00	Normal	/	r.906C>T; p.(Tyr302=)	Heterozygous variant in RNA	PM2, BP7_S	2
CHEK2	c.846+4_846+7del	p.?	Alteration of the consensus splice site 98.41% [91.47%-99.96%]	AG: 0.00; AL: 0.00; DG: 0.00; DL: 0.98 (8)	Exons 7_8 skipping + exon 7 skipping	Confirmed by RT-PCR Confirmed in bibliography (30,31)	r.793_908del; p.(Asp265Alafs∗7) r.793_846del; p.(265_282del)	/	PM2, PVS1, PS3	5
CHEK2	c.538C>T	p.(Arg180Cys)	Alteration of an exonic splicing regulatory element 35.81% [28.11%-44.1%]	AG: 0.00; AL: 0.00; DG: 0.00; DL: 0.00	Normal	Confirmed by RT-PCR	r.538C>T; p.(Arg180Cys)	/	PM2, BP7_NA	3
FLCN	c.-113-1G>A	p?	Alteration of the consensus splice site 98.41% [91.47%-99.96%]	AG: 0.42; AL: 0.95; DG: 0.00; DL: 0.00	Exon 3 skipping (5'UTR)	/	r.-113_-25del; p.?	/	PM2, PVS1_NA	3
MLH1	c.791-489_791-20del	p.?	Alter BP + alter by creating cryptic 36.17% [26.46%-45.88%]	NA	Partial exon 10 skipping	RT-PCR : partial exon 10 skipping Mini-gene : total exon 10 skipping	r.791_884del; p.(His264Leufs∗2)	No heterozygous variant	PM2, PVS1_S, PP4, PP1	4
MLH1	c.306+5G>T	p.?	Alteration of the consensus splice site 98.41% [91.47%-99.96%]	AG: 0.00; AL: 0.00; DG: 0.12; DL: 0.79	Cryptic donor site creation	RT-PCR: partial effect Mini-gene: total effect	r.302_306del; p.(Glu102Phefs∗18)	No heterozygous variant	PM2, PVS1, PM5	5
MLH1	c.882C>G	p.(Leu294=)	Alteration of the consensus splice site 85.91% [79.27%-91.06%]	AG: 0.00; AL: 0.00; DG: 0.00; DL: 0.05	Partial exon 10 skipping	Partial effect confirmed by RT-PCR Partial effect confirmed by mini-gene in bibliography (12)	p.?	/	PM2, PVS1_NA	3
MLH1	c.117-16_117-15del	p.?	Alteration of the polypyrimidine tract (-20 to -18) 23.61% [16.94%-31.4%)	AG: 0.00; AL: 0.02; DG: 0.00; DL: 0.00	Normal	/	r.=	Heterozygous variant in RNA	PM2, BP7_S	2
MLH1	c.1897-42C>T	p.?	Alteration of the branch point 43.04% [35.2%-51.14%] AG:0.03; AL:0.00;DG:0.00;DL:0.00	AG: 0.03; AL: 0.00; DG: 0.00; DL: 0.00	Normal	/	r.=	Heterozygous variant in RNA	PM2, BP7_S	2
MSH2	c.793G>A	p.(Val265Ile)	Alteration of the consensus splice site 35.81% [28.11%-44.1%]	AG: 0.03; AL: 0.00; DG: 0.00; DL: 0.00	Normal	Confirmed by RT-PCR	r.793G>A; p.(Val265Ile)	Heterozygous variant in RNA	PM2, BP7_NA	3
MSH6	c.3537C>G	p.(Ala1179=)	NTR 08.25% [04.79%-13.05%]	AG: 0.00; AL: 0.00; DG: 0.00; DL: 0.00	Normal	Confirmed by RT-PCR	r.3537C>G; p.(Ala1179=)	Heterozygous variant in RNA	PM2, BP7_S	2
MSH6	c.3173-22C>G	p.?	Alter BP 13.87% [08.56%-20.81%]	AG: 0.01; AL: 0.03; DG: 0.00; DL: 0.00	Normal	/	r.=	Heterozygous variant in RNA	PM2, BP7_S	2
MSH6	c.153C>T	p.(Ser51=)	NTR 03.43% [01.39%-06.94%]	AG: 0.00; AL: 0.00; DG: 0.00; DL: 0.00	Normal	/	r.153C>T; p.(Ser51=)	Heterozygous variant in RNA	PM2, BP7_S	2
MUTYH	c.1103-27C>T	p.?	Alter BP 98.11% [94.59%-99.61%]	AG: 0.01; AL: 0.00; DG: 0.00; DL: 0.00	Normal	/	r.=	Heterozygous variant in RNA	PM2, BP7_S	2
NF1	c.731-8del	p.?	Alteration of the consensus splice site 23.61% [16.94%-31.4%]	AG: 0.00; AL: 0.00; DG: 0.00; DL: 0.00	Normal	/	r.=	No heterozygous variant	PM2, BP7_NA	3
NF1	c.2252-16del	p.?	NTR 05.74% [02.81%-11.37%]	AG: 0.00; AL: 0.03; DG: 0.00; DL: 0.00	Normal	/	r.=	Heterozygous variant in RNA	PM2, BP7_S	2
NF2	c.1122+6T>C	p.?	Alteration of the consensus splice site 98.41% [91.47%-99.96 %]	AG: 0.00; AL: 0.00; DG: 0.00; DL: 0.01	Normal	Confirmed by RT-PCR	r.=	No heterozygous variant	PM2, BP7_NA	3
NF2	c.1000-7C>G	p.?	Alteration of the consensus splice site 30.67% [23.41%-38.71%]	AG: 0.00; AL: 0.00; DG: 0.00; DL: 0.00	Normal	/	r.=	No heterozygous variant	PM2, BP7_NA	3
PALB2	c.2379C>T	p.(Gly793=)	NTR 03.48% [01.41%-07.04%]	AG: 0.01; AL: 0.00; DG: 0.66; DL: 0.00	Normal	/	r.2379C>T; p.(Gly793=)	Heterozygous variant in RNA	PM2, BP7_S	2
PMS2	c.23+1G>T	p.?	Alteration of the consensus splice site 98.41% [91.47%-99.96%]	AG: 0.00; AL: 0.00; DG: 0.37; DL: 0.99	Intronic retention	RT-PCR failure (pseudogene) Tumor PMS2 loss	r.?; p.?	No heterozygous variant	PM2, PVS1, PP4	5
PMS2	c.1004A>G	p.(Asn335Ser)	NTR 09.76% [06.06%-14.67%]	AG: 0.00; AL: 0.00; DG: 0.01; DL: 0.00	Normal	/	r.1004A>G; p.(Asn335Ser)	Heterozygous variant in RNA	PM2, BP7_NA	3
PMS2	c.803+5G>A	p.?	Alteration of the consensus splice site 98.41% [91.47%-99.96%]	AG: 0.00; AL: 0.00; DG: 0.01; DL: 0.98	Partial crytic acceptor site use (r.762_803del)	Confirmed by RT-PCR	r.?; p.?	No heterozygous variant	PM2, PVS1_NA	3
POLE	c.5678+6G>A	p.?	Alteration of the consensus splice site 30.67% [23.41%-38.71%]	AG: 0.00; AL: 0.00; DG: 0.00; DL: 0.00	Normal	Confirmed by RT-PCR	r.=	Heterozygous variant in RNA	PM2, BP7_S	2
PTEN	c.209+6T>G	p.?	Alteration of the consensus splice site 98.54% [94.83%-99.82%]	AG: 0.00; AL: 0.77; DG: 0.00; DL: 0.85	Exon 3 skipping	Confirmed by RT-PCR and mini-gene	r.165_209del; p.(Arg55_Leu70delinsSer)	No heterozygous variant	PM2, PVS1	4
RAD51C	c.1026+5_1026+7del	p.?	Alteration of the consensus splice site 98.54% [94.83%-99.82 %]	AG: 0.00; AL: 0.00; DG: 0.09; DL: 0.88	Exon 8 skipping	Confirmed by RT-PCR	r.966_1026del; p.(Arg322Serfs∗22)	No heterozygous variant	PM2, PVS1, PP5	5
RAD51C	c.513C>T	p.(Asp171=)	NTR 04.35% [02.01 %-08.09%]	AG: 0.00; AL: 0.00; DG: 0.00; DL: 0.00	Normal	/	r.513C>T; p.(Asp171=)	Heterozygous variant in RNA	PM2, BP7_S	2
SDHA	c.762_770+17del	p.(Ala255_Gly257del)	Alteration of the consensus splice site + alteration of an exonic splicing regulatory element 98.41% [91.47%-99.96%]	NA	Multiple complex alterations	/	r.?; p.?	/	PM2, PVS1	4
SDHB	c.541-27T>G	p.?	NTR 09.76% [06.06%-14.67%]	AG: 0.00; AL: 0.00; DG: 0.00; DL: 0.00	Normal	/	r.=	No heterozygous variant	PM2, PVS1_NA	3
SDHC	c.19A>G	p.(Arg7Gly)	+ Alteration of the consensus splice site + alteration of an exonic splicing regulatory element 98.41% [91.47%-99.96%]	AG: 0.00; AL: 0.00; DG: 0.01; DL: 0.66	Partial intronic retention		r.?; p.?	/	PM2, PVS1_M	3
TMEM127	c.-112G>T	p.?	NTR 04.63% [02.81%-07.14%]	AG: 0.01; AL: 0.17; DG: 0.00; DL: 0.00	Normal	/	r.=	No heterozygous variant	PM2, PVS1_NA	3

Abbreviations: AG: acceptor gain; AL: acceptor loss; DG: donor gain; DL: donor loss; NTR: nothing to report; BP: branch point; AT: ataxia telangiectasia [12, 29–31].

**Table 3:** Performance comparison of SpIP and SpliceAI tools in predicting abnormal splicing.

		RNA analysis			
Software	Prediction	Total splice effect	Partial splice effect	No splice effect	Total
SpIP predictions	Positive > 50%	11	4	4	19
	Positive < 50%	1	1	15	17
	NTR	0	0	14	14
	NA	1	2	0	3

SpliceAI predictions	Positive > 0.50	10	1	1	12
	Positive < 0.50	1	1	2	4
	Negative < 0.20	1	1	30	32
	NA	1	4	0	5
Total		13	7	33	53

Abbreviation: NTR: nothing to report.

**Table 4:** Classification of large duplications by the targeted RNA panel.

Gene	Reference	Variant	Exons	Chimeric reads	Variant classification
ATM	NM_000051.4	c.(8850+1_8855-1)_(∗1_?)dup	62-63	No	3
ATM	NM_000051.4	c.(2466+1_2467-1)_(8850+1_8851-1)dup	17-61	Yes	5
MSH2	NM_000251.3	c.(1076+1_1077-1)_(1276+1_1277-1)dup	7	Yes	5
PMS2	NM_000535.7	c.(1144+1_1145-1)_(2174+1_2175-1)dup	11-12	Yes	5

## Data Availability

Most of the data analyzed during this study can be found within the published article and its tables. Any additional raw data are available from the corresponding author on reasonable request.
